# Neuronal representation of working memory in the medial prefrontal cortex of rats

**DOI:** 10.1186/s13041-014-0061-2

**Published:** 2014-08-28

**Authors:** Sheng-Tao Yang, Yi Shi, Qi Wang, Ji-Yun Peng, Bao-Ming Li

**Affiliations:** 1 Institute of Neurobiology & State Key Laboratory of Medical Neurobiology, Fudan University, Shanghai 200032, China; 2 Center for Neuropsychiatric Diseases, Institute of Life Science, Nanchang University, Nanchang 330031, China

**Keywords:** Neuron activity, Medial prefrontal cortex, Working memory, Rats

## Abstract

Working memory is a process for short-term active maintenance of information. Behavioral neurophysiological studies in monkeys have demonstrated that the dorsolateral prefrontal cortex (dlPFC) is a key cortical region for working memory. The medial prefrontal cortex (mPFC) in rats is a cortical area similar to the dlPFC in monkeys in terms of anatomical connections, and is also required for behavioral performance on working-memory tasks. However, it is still controversial regarding whether and how mPFC neurons encode working memory. In the present study, we trained rats on a two-choice spatial delayed alternation task in Y maze, a typical working memory task for rodents, and investigated neuronal activities in the mPFC when rats performed the task. Our results show that, (1) inactivation of the mPFC severely impaired the performance of rats on the task, consistent with previous studies showing the importance of the mPFC for working-memory tasks; (2) 93.7% mPFC cells (449 in 479) exhibited changes in spiking frequency that were temporally locked with the task events, some of which, including delay-related cells, were tuned by spatial information; (3) differential delay activities in individual mPFC cells appeared transiently and sequentially along the delay, especially during the early phase of the delay; (4) some mPFC cells showed no change in discharge frequency but exhibited differential synchronization in firing during the delay. The present results suggest that mPFC neurons in rats are involved in encoding working memory, via increasing firing frequency or synchronization.

## Introduction

Working memory is a short-term memory system for active maintenance and manipulation of information in order to guide behavior, and is considered as a core function of the prefrontal cortex (PFC) [1]–[4]. Lesion, electrophysiological in monkeys and imaging studies in humans have well demonstrated that the dorsolateral PFC (dlPFC) is a critical cortical area for working memory [4]–[8].

The most significant difference in the PFC between rodents and primates is that, while the primate prefrontal cortex has obvious granular layer, known as layer IV, the rodent prefrontal cortex has no obvious granular layer. However, studies on neural projections and functions of the prefrontal cortical subareas support a view that the medial PFC (mPFC), also termed prelimbic cortex (PrL), in rodents is similar to the dlPFC in monkeys (for review see Seamans et al.) [9].

It has been documented that the rat mPFC is required for working-memory task performance. For example, lesion to the mPFC impairs the performance of rats on delayed alternation tasks [10]–[12]. Thus, there should be cells in the mPFC that encode working memory. However, previous studies showed that few neurons in the mPFC exhibited differential delay discharge when rats performed working-memory tasks. For example, Jung et al. [13],[14] recorded neuronal activities in the mPFC when rats performed an eight-arm radial maze task or a Table 1 maze task. They found that mPFC neurons increased firing during the delay, but few cells discharged differentially. Baeg et al. [15] reported that cell assemblies in the mPFC could predict spatial locations of delayed choice in the Table 1 maze, but majority of the cells making-up of the assemblies were not delay cells.

**Table 1:** Summary of multi-event related neurons in the medial prefrontal cortex

Delay-related cells (n=259)	D	DC	DR	DB	DCR	DCB	DRB	DCRB
	40	23	49	40	8	24	41	34
Choice related cells (n=152)	C	DC	CR	CB	DCR	DCB	CRB	DCRB
	16	23	18	16	8	24	13	34
Reward related cells (n=264)	R	DR	CR	RB	DCR	DRB	CRB	DCRB
	74	49	18	27	8	41	13	34
Running back related cells (n=221)	B	DB	CB	RB	DCB	DRB	CRB	DCRB
	26	40	16	27	24	41	13	34
Delay cells: differential (n=47)	D	DC	DR	DB	DCR	DCB	DRB	DCRB
	19	2	10	5	2	1	4	4
Choice cells: differential (n=58)	C	DC	CR	CB	DCR	DCB	DRB	DCRB
	12	2	14	12	2	1	11	4
Reward cells: differential (n=132)	R	DR	CR	RB	DCR	DRB	CRB	DCRB
	61	10	14	26	2	4	11	4
Running back cells: differential (n=76)	B	DB	CB	RB	DCB	DRB	CRB	DCRB
	13	5	12	26	1	4	11	4

**Figure 1:**
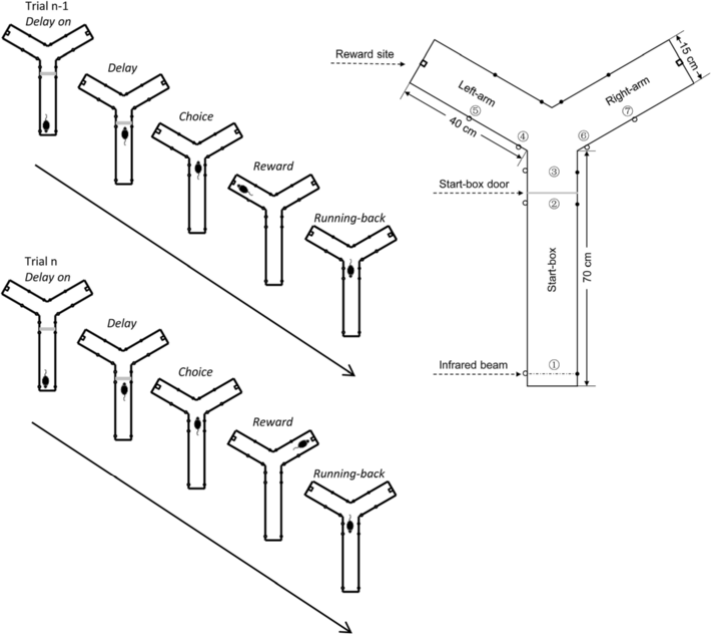
**Delayed alternation task in Y-maze.** The Y-maze consisted of a start box and two arms (left and right). Inside the maze were installed seven infrared beams, marked by #1 through #7. Each trial was started by rats’ breaking the infrared beam #1 in the start box. After a delay of 6 seconds, the start-box door was opened and rats were allowed to visit the two arms. If rats had chosen the left arm in the previous trial, they were required to select the right arm in the present trial, or *vice versa*. Thus, rats had to remember the arm they had visited in the previous trial in order to make a correct choice in the present trial. After consuming the water reward at the reward site, rats ran back to the start box to initiate the next trial.

It was possible that the eight-arm radial maze task was too complex and too difficult to address working-memory cells in rodents, whereas the Table 1 maze did not include a forced inter-trial delay and rats might establish an automatic running behavior between the two opposite directions in the maze. To reexamine the cellular representation of working memory in the mPFC, the present study employed a Y-maze alternation task with a forced inter-trial delay (Figure 1). In this task, rats were restrained in the start box of the maze during a delay period of 6 seconds. After the delay, rats were allowed to choose entering the left- and right-arm of the maze alternatively. After consuming water reward, rats ran back to the start box and a second delay was forced. Because of the forced delay between consecutive trials, it was less possible for the animals to develop automatic motor behavior. By using this paradigm, we successfully identified differential delay cells in the mPFC, which seem to represent working memory for spatial locations.

## Results

### Performance of the delayed alternation task

A total of 27 rats were used, of which 9 rats for mPFC inactivation experiments and 18 rats for neuronal recording experiments. After the animals performed the task with correct rate of > 80% in two successive sessions, they were used for mPFC inactivation or electrophysiological recording. For electrophysiological recording experiments, a total of 126 daily sessions were recorded, and the animals performed 135.13 ± 4.03 trials (mean ± sem) in a daily session, with correct rate of performance of 94.67% ± 0.66%.

### Inactivation of mPFC impairs task performance

**Figure 2:**
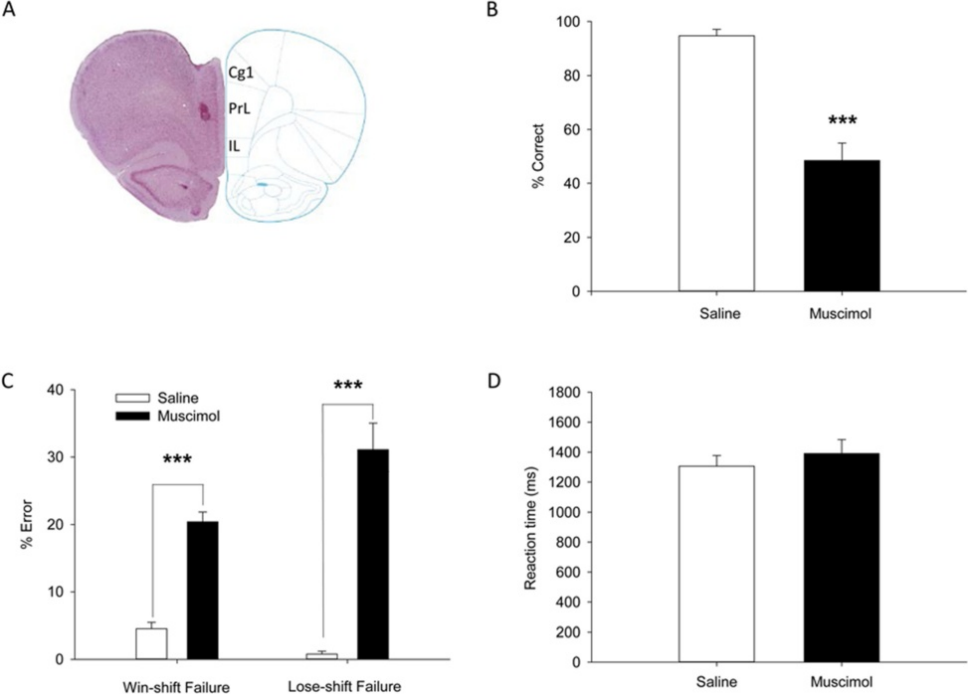
**Intra-mPFC infusion of muscimol impairs the performance of rats on the delayed alternation task. (A)** Infusion sites of muscimol in the mPFC, as indicated by the arrow. **(B)** The correct rate of task performance was reduced upon muscimol infusion. **(C)** The win-shift and lose-shift strategies were destroyed after muscimol treatment, with a significant increase in win-shift and lose-shift failures. **(D)** Reaction time (RT) was unchanged upon muscimol infusion. RT was defined as the duration from the delay off to the breaking of infrared beam #4 or #6. ***p < 0.001, n = 9 rats, paired *t*-test. Cg1, cingulate cortex, area 1; IL, infralimbic cortex; PrL, prelimbic cortex.

To investigate the role of mPFC in the task performance, we bilaterally infused the GABA_A_ receptor agonist muscimol into the mPFC (each side 0.5 μg/0.5 μL; 8.8 mMol/L). Saline was similarly infused as vehicle control (each side 0.5 μL). Infusions of muscimol or saline were made at the central mPFC (AP +3.5 mm to bregma). Histological examination with neutral red staining of brain sections confirmed the location of infusion (Figure 2A). As shown in Figure 2, inactivation of the mPFC severely impaired animals’ performance on the delayed-alternation task (Figure 2B; p < 0.001, n = 9, paired *t*-test). Analysis of error types revealed that, the animals made significantly more win-shift and lose-shift failures upon inactivation of the mPFC (Figure 2C; p < 0.001 vs saline, n = 9 rats, paired *t*-test), with the reaction time unaffected (Figure 2D; p = 0.17, n = 9, unpaired *t*-test). This result suggests the importance of the mPFC not only for working-memory performance but also for error correction, as win-shift strategy may reflect the capability of rats to use working memory, whereas the lose-shift strategy reflects the ability of rats to correct errors.

### Database of task-related mPFC cells

**Figure 3:**
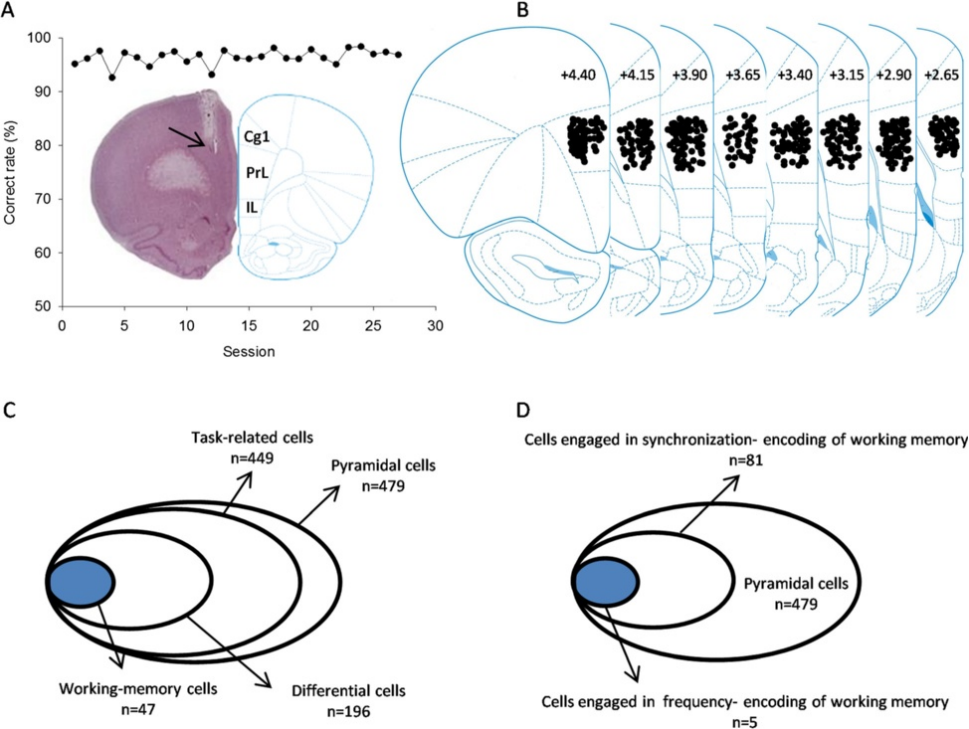
**Database of mPFC neurons recorded when rats performed the delayed alternation task. (A)** Stable performance of a rat during 27 recording sessions. Shown in insert is the placement site of electrode in the mPFC, as indicated by the arrow. **(B)** The placement sites of electrode in the mPFC. **(C)** The database of pyramidal cells sampled and analyzed. **(D)** The database of cells engaged in working-memory encoding. Cg1, cingulate cortex, area 1; IL, infralimbic cortex; PrL, prelimbic cortex.

Spike recording was performed at from AP 2.5-4.5 mm in the mPFC. The animals showed a stable performance on the task during the recording sessions (Figure 3A). A total of 479 pyramidal cells and 26 interneurons were recorded and isolated from the mPFC. Putative pyramidal cells and interneurons were distinguished by their waveforms and firing frequency [13]. Due to the small number of interneurons (26 in 505, 5.1%), we only focused on the pyramidal cells in the present study. Figure 3B,C, D and Table 1 shows the database of the task-related cells. The rank-sum test was used in individual cells to identify task-related firing. If p value was <0.01 in three successive bins (100 ms bin, 50 ms step) during a task period, we considered the cell as being task-related.

Among the 479 pyramidal cells isolated, 449 showed change in firing frequency during the task performance (frequency-encoding cells). Many frequency-encoding cells exhibited activities related to multiple events of the task (for detail see Table 1). Of the 449 task-related cells, 196 showed preference or differential firing for left or right trials, among which 47 were delay cells (defined as working-memory cells), 58 choice cells, 132 reward cells, and 76 running-back cells. In addition, we have identified 94 cell pairs, from 81 cells, which were engaged in synchronization encoding of working memory.

### Frequency encoding of working memory

**Figure 4:**
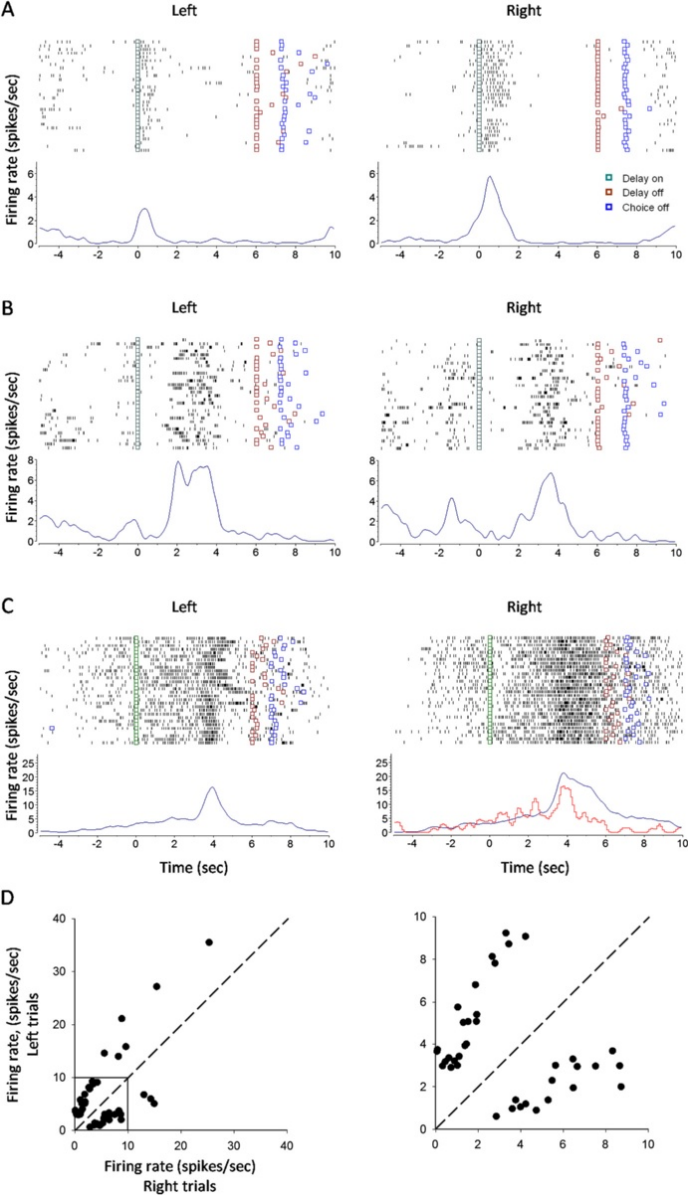
**mPFC neurons with differential discharge during the early, middle and late delay. (A)** An early-delay neuron with firing preference for right trials (p < 0.01 for right vs. left trials; rank-sum test). **(B)** A middle-delay neuron with firing preference for left trials (p < 0.01 for left vs. right trials). **(C)** A late-delay neuron with firing preference for right trials (p < 0.01 for right vs. left trials). Red line: error trials. **(D)** Plotting of firing rates during left-trial delay against those during right-trial delay for the differential delay neurons (n = 47). Shown in the right panel is the enlargement of the square box in the left panel.

**Figure 5:**
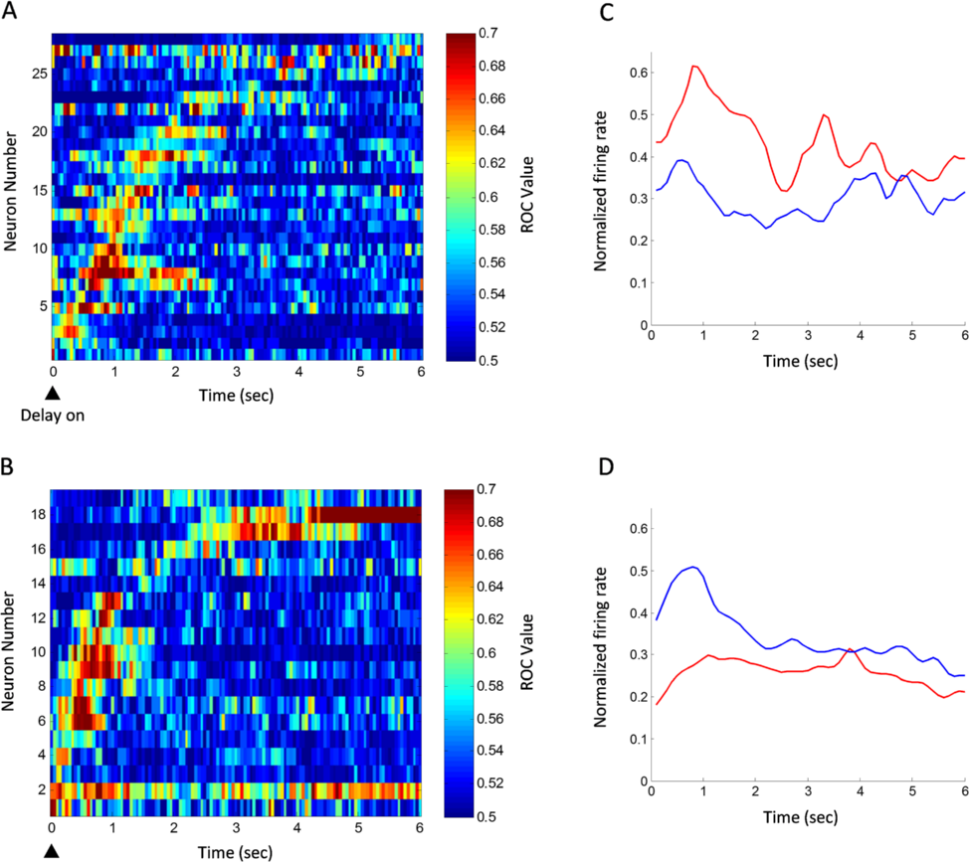
**Temporal evolutions of the spatial preference of differential delay neurons. (A)** The ROC values of neurons with preference for left trials. **(B)** The ROC values of neurons with preference for right trials. Each raster represents the ROC value of an individual cell, which was aligned with the onset of the delay period. The cells were sorted by time when their ROC values first reached 0.61 in three consecutive bins (100 ms bin, 50 ms step). The colors in each grid represented ROC values. **(C)** and **(D)** are the average PSTH of neurons with preference for left and right trials, respectively. The normalized firing rate (Fn) is calculated by the formula: Fn = (F-Min)/(Max-Min), where F is the firing rate of neurons, Min and Max are the minimum and maximum of the firing rate, respectively. Red line: left trial; blue line: right trial.

A total of 259 delay-related cells were identified, of which 47 showed firing preference for left trials (n = 28) or right trials (n = 19). All of the delay-related cells demonstrated a transient but not persistent discharge during the delay. Figure 4 shows three cells with differential discharge during the early (A), middle (B) and late delay (C). Figure 4A is an early-delay cell with discharge preference for right trials (p < 0.01 for right vs. left trials; rank-sum test), Figure 4B a middle-delay cell having activity preference for left trials (p < 0.01 for left vs. right trials), and Figure 4C a late-delay cell with firing preference for right trials (p < 0.01 for right vs. left trials). Interestingly, the differential delay discharge disappeared in trials with incorrect choice (see the red line in Figure 4C, right). Figure 4D shows the plotting of the firing rates during left-trial delay against those during right-trial delay for the differential delay neurons (n = 47). Receiver operating characteristic (ROC) analysis revealed that the activities of these cells appeared sequentially during the delay (Figure 5A and B). Figure 5C and D show the average peristimulus time histograms (PSTH) of the differential delay neurons. Thus, there exist cells in the mPFC of rats that are involved in encoding working memory by increasing firing frequency.

### Synchronization encoding of working memory

To investigate synchronization encoding of working memory, we adopted joint perievent time histogram (JPETH) to depict cross-correlation of cell pairs recorded simultaneously. The larger the cross-correlation index [−1, +1] was, the higher the cell-cell synchronization [16]. We have identified 94 pairs of cells, from a total of 81 individual cells, which did not show any change in discharge frequency but exhibited strong synchronization in firing during the delay, suggesting that some cells in the mPFC are dynamically organized into functional assembly during the delay.

**Figure 6:**
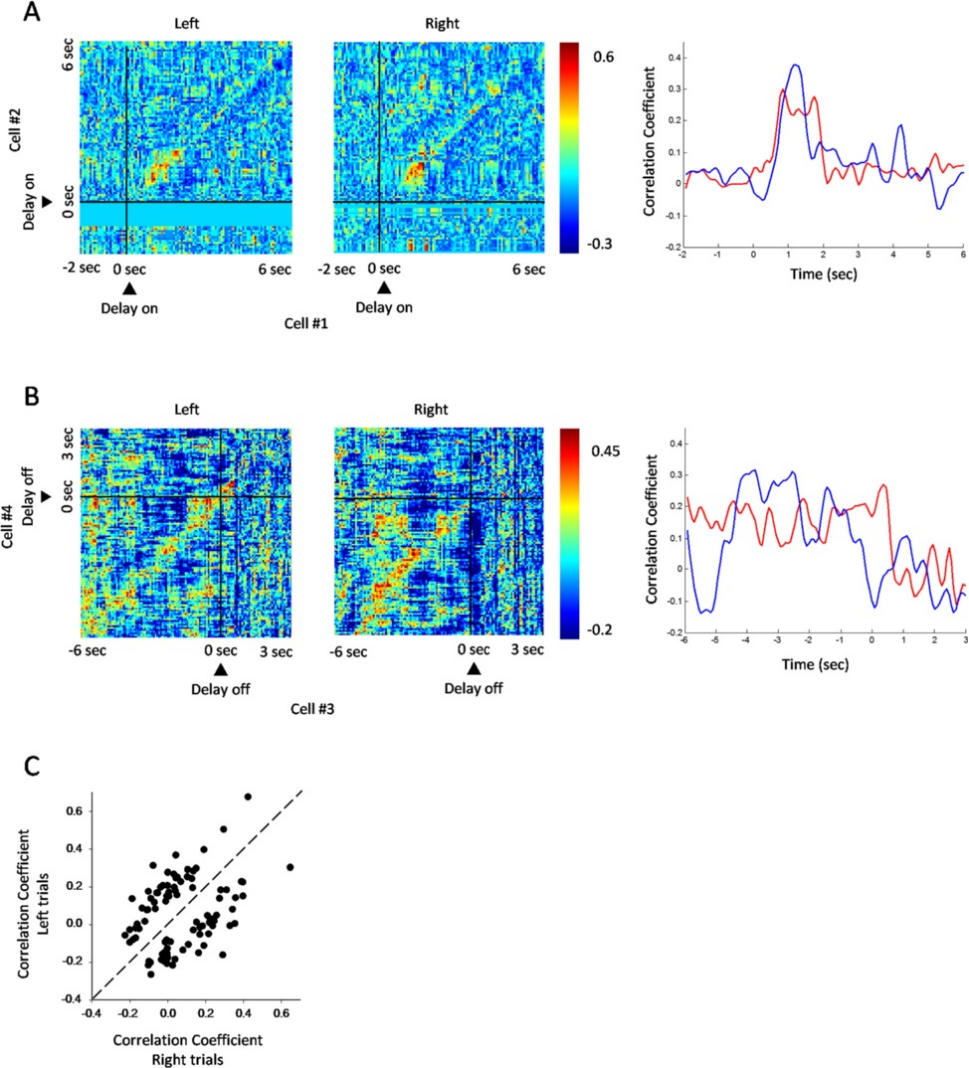
**Synchronized activities of mPFC cell pairs during the delay period. (A)** Spiking synchronization of a cell pair during the early delay. The left and middle panels were JPETH of the cell pair in left and right trials, respectively (75 ms bin), aligned with delay on. This cell pair showed shorter but stronger correlation in right trial than in left trial (p < 0.05; two-tailed *t*-test). Shown in the right panel were the correlation coefficients along main diagonal of the JPETH. Red line: left trial; blue line: right trial. **(B)** Spiking synchronization of a cell pair during the middle delay. The left and middle panels were JPETH of the cell pair in left and right trials, respectively (75 ms bin), aligned with delay off. This cell pair displayed shorter but stronger correlation in right trial than in left trial (p < 0.05; two-tailed *t*-test). Shown in the right panel were the correlation coefficients along main diagonal of the JPETH. Red line: left trial; blue line: right trial. **(C)** Plotting of correlation coefficients during left-trial delay against those during right-trial delay for individual cell pairs (n = 94 cell pairs). Some cell pairs showed synchronization preference for left trial, and others for right trials.

Figure 6 shows two pairs of cells which demonstrated synchronized activity during the early (A) and middle delay (B), with shorter but stronger correlation in the right vs. left trials (p < 0.05; two-tailed *t*-test), suggesting that the synchronized firing of the cell pairs were tuned by working memory. Figure 6C shows the plotting of the correlation coefficients during left-trial delay against those during right-trial delay (n = 94 cell pairs). As shown, some cell pairs showed synchronization preference for left trial, while others for right trial. Thus, mPFC cells are involved in encoding working memory by increasing firing synchronization.

### Other types of task-related activities

**Figure 7:**
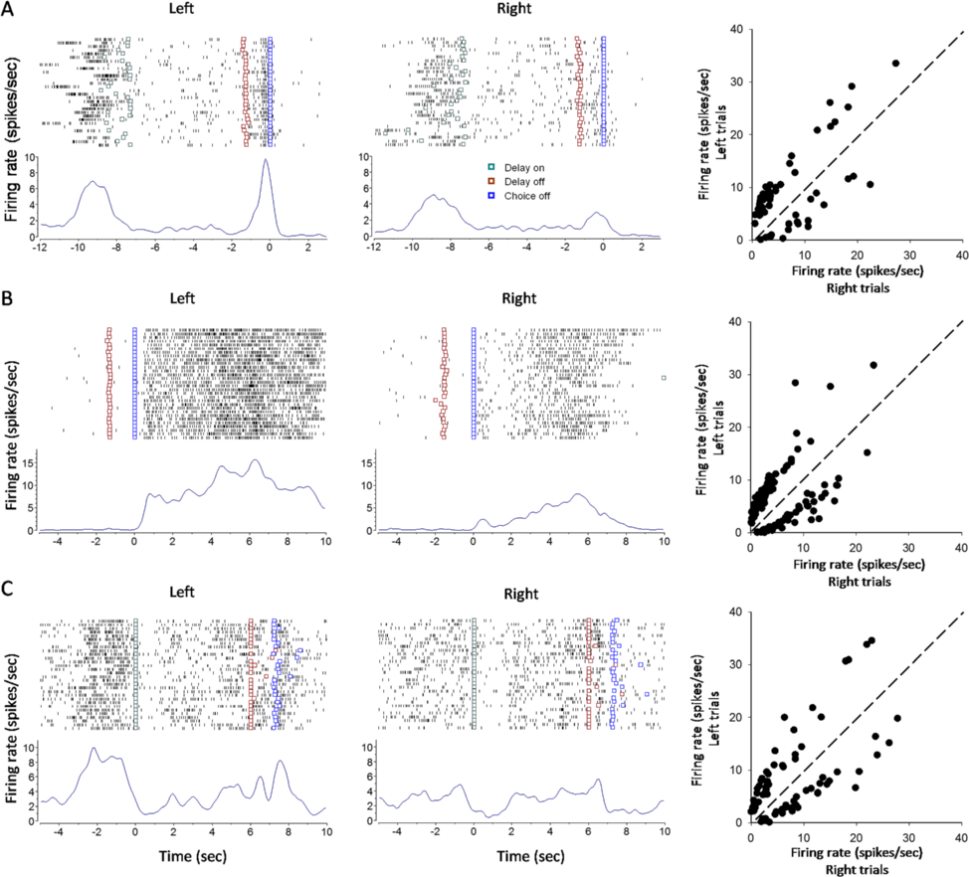
**Task-related activities with spatial preference during the choice, reward and running-back periods. (A)** A choice cell having preference for left trials (p < 0.01 for left vs. right trials; rank-sum test). Shown in the right panel is the plotting of firing rates during the choice period in left trials against those in right trials. n = 58 differential choice cells. **(B)** A reward cell having preference for left trials (p < 0.01 for left vs. right trials; rank-sum test). Shown in the right panel is the plotting of firing rates during reward in left trials against those in right trials. n = 132 differential reward neurons. **(C)** A running-back cell having preference for right trials (p < 0.01 for right vs. left trials; rank-sum test). Shown in the right panel is the plotting of firing rates during the running-back period in left trials against those in right trials. n = 76 differential running-back neurons.

A quite many cells in the mPFC demonstrated a change in firing frequency during the choice (n = 58 cells), reward (n = 132 cells), and running-back periods (n = 76 cells). Interestingly, differential discharge was also observed in these cells. Figure 7A is a choice cell having preference for left trials (p < 0.01 for left vs. right trials; rank-sum test), Figure 7B a reward cell with preference for left trials (p < 0.01 for left vs. right trials), and Figure 7C a running-back cell with preference for right trials (p < 0.01 for right vs. left trials). Persistent and differential firing could be seen during the reward and running back periods (Figure 7B and C). It seems that these cells were tuned by trial-based spatial information.

## Discussion

The present results suggest that mPFC neurons in rats are involved in encoding working memory, via increasing firing frequency or synchronization during the delay period.

### Working memory tasks for monkeys and rodents

There have been a few classical working memory paradigms for monkeys, such as oculomotor delayed response (ODA) and delayed matching-to-sample (DMS) tasks. In these tasks, monkeys are required to remember a spatial or feature cue and make a corresponding response or choice after a delay period [1],[3]. For rodents, Y- or T-maze, eight-arm maze and Table 1 maze have been employed quite often [13],[17],[18], whereby rodents are required to remember their previous choice and shift their choice in the present trial. The key difference is that, while the monkey’s tasks have a temporally controlled and forced delay interposed between the cue and response choice, the rodent’s ones usually do not.

Without a controlled and forced delay, it might be easy for animals to develop, by repeated training and performance over trials, an automatic alternation or habit behavior in the Y- or T-shape mazes, and especially in the Table 1 maze, where rats ran in a Table 1 path. Thus, working memory may be dispensable for the performance of these tasks. To address neuronal representation of working memory in the rodent mPFC, the present study modified the Y-maze paradigm by interposing a delay of 6 seconds between consecutive trials. It should be noticed that, in this modified version of Y-maze task, the actual delay in each trial was longer than 6 seconds, and was flexible in length, considering that the rats spent a few seconds on running-back to the start box. Moreover, the rats had to run back to the end of the start box and breaking the infrared beam #1 to initiate each trial (see Figure 1). Thus, it was less possible for the animals to develop an automatic alternation strategy, but use working memory, to perform the task.

### Importance of the mPFC for working memory task

Many previous studies, especially those in non-human primates, have shown that the prefrontal cortex is the functional center for working memory [19],[20]. In the present study, we found that local inactivation of the mPFC of rats with muscimol severely impaired the win-shift performance strategy of the animals, suggesting that working memory for a just- or to-be-visited location/direction (left- or right-arm) might be interrupted. This is consistent with previous lesion or inactivation studies in rodents and non-human primates showing the critical role of the prefrontal cortex in working memory performance [11],[12],[19]. Moreover, the lose-shift performance strategy, that is, the error-correcting ability, was also severely destroyed upon inactivation of the mPFC: the animals re-entered an arm where they had visited and was not rewarded in the previous trial.

Inactivation by muscimol has been widely used to address functional importance of cortical or subcortical structures [21]–[23]. The disadvantage of this method is that, muscimol produces non-specific effect on different types of neurons, and the inactivation is not temporally controlled. Thus, intra-mPFC infusion of muscimol may have a direct effect on working memory *per se* through inactivating delay activity, or affect the task performance by inactivating neuronal activity in the choice, reward or running-back period.

### Characteristics of task-related mPFC cells

Several characteristics can be summarized for the task-related activities in the rat mPFC. Firstly, most of the task-related cells are multi-event related cells, that is, an individual cell could be involved in different task events (Table 1). The multi-event involvement may facilitate information transfer across task periods. Secondly, all of the delay cells, either differential or non-differential ones, showed transient but not persistent activity, which is different from dlPFC cells in monkeys exhibiting sustained firing during delay [1]. Thirdly, differential and non-differential firing existed in all of the task periods. It was possible that the differential activities reflect trial-type effects on cells, such as spatial information or working memory, while the non-differential activities reflect general task components, such as motor running, reward expectation, and reward consuming. Fourthly, the transient and differential delay activities in individual cells appeared at different temporal points during the delay. The sequential firing pattern during the delay may reflect relay-race transfer of working memory information [24]. The firing difference between the rodent mPFC (transient activity) and the monkey dlPFC (persistent activity) may reflect the difference in evolutional hierarchy of the two species.

### Encoding of mPFC cells for working memory

The present study showed that, a portion of delay-related mPFC cells, despite limited number, were tuned by spatial information. Some cells exhibited firing preference for left trial, while others for right trial. These differential delay-related cells may be engaged in encoding the spatial information. It might be possible that these cells were encoding the location where the animals were to visit (prospective encoding), or where the animals had just visited (retrospective encoding). Indeed, if a delay-related cell with differential activity exhibited a firing pattern that was not for the preferred but non-preferred direction, an error behavioral choice could be well predicted (Figure 4C).

There are anatomical and functional differences between rodent mPFC and primate dlPFC [25]. Based on developed criteria on homologous structure, Uylings et al. [26] suggest that the rodent mPFC is similar to the primate dlPFC [26]. In the present study, we failed to encounter cells in the mPFC with sustained firing during the delay period. Most of the delay-related cells showed a transient increase in discharge. ROC analysis revealed that, the delay-related activities appeared sequentially, suggesting that mPFC cells may carry on working-memory information by sequential activation for information flow among cells. This is well consistent with the previous studies in monkeys showing that some dlPFC neurons demonstrate sequential activation during delay [27],[28].

Some cell pairs in the mPFC fired in synchronized way during the delay period, although they did not show any change in firing frequency. Such synchronization was also tuned by spatial information. This result suggests that, mPFC cells are dynamically and functionally organized together to encode working-memory information. Using cross-correlation analysis, Funahashi and Inoue [27] observed synchronized firing among dlPFC neurons of monkeys performing a working-memory task.

Inconsistent with the results by Jung et al. [13], the present study identified differential delay cells in the mPFC. It should be pointed out that, the eight-arm radial maze and the Table 1 maze used by Jung et al. may not be the best paradigms for accessing working memory cells in the mPFC. Probably, the eight-arm radial maze task was too complex and too difficult to address working memory cells in rodents, whereas the Table 1 maze did not include a forced delay, and rats might establish a kind of habit to run alternatively between the two opposite arms.

### Functional significance of choice-related mPFC cells

The choice period, especially the early stage of the choice period, was a task period when the animals decided where to go. Working-memory information might be processed and transformed to action-executing information during this period. In the present study, some mPFC cells demonstrated a differential discharge during the choice period. It was possible that these cells were still carrying on working memory information. Takeda and Funahashi [29] reported that, information encoded by dlPFC neurons in monkeys changed from visual to acting information during late delay. This view of information transformation was also supported by other studies [28],[30].

### Functional significance of reward-related mPFC cells

The animals received water reward in the maze arms after a correct choice. Interestingly, some mPFC cells demonstrated a differential and persistent discharge even during this period (see Figure 7B). The differential reward-related activity could not be explained as due to reward-consuming movements, or water reward *per se* (such as quality and amount), as the animals executed similar movements for consuming reward, and got equal amount of water after a correct choice in both left- and right-arm trials. Rather, it might reflect a kind of mental status for evaluating the correct behavioral choice. Jung et al. [13] also reported that mPFC neurons exhibited differential reward activity in the eight-arm maze and Table 1 maze tasks.

### Functional significance of running-back related cells

The present study interposed a delay of 6 seconds after the animals returned into the start box. However, the running-back period could be considered as a period of the total inter-trial delay in each trial. The animals ran back to the start box after they had consumed the water reward, a task period when spatial information for a visited arm might be maintained in the mPFC. The present study found that, some mPFC neurons displayed a differential and even persistent discharge during the running-back period (see Figure 7C). These neurons might be retrospectively encoding the spatial information for a just-visited arm, a kind of representation for spatial working memory. Consistently, differential running-back discharge was also observed in the mPFC of rats performing the Table 1 maze task [13].

## Conclusion

The present study shows that individual neurons in the mPFC of rats are involved in representing working memory by increasing firing frequency or synchronization, providing electrophysiological evidence that the rat mPFC is functionally similar to the monkey dlPFC.

## Materials and Methods

### Animals

Male Spraque-Dawley rats (8–10 weeks old; 250–350 g) were used. Rats were purchased from the Shanghai Laboratory Animal Center, Chinese Academy of Sciences (Shanghai, China). They were housed 2–4 per cage under constant temperature (23 ± 1°C) and light-controlled vivarium (12 h light/12 h dark cycles). Food and water were available ad libitum. Surgery was executed after habituation of 7 days to our laboratory vivarium. All experimental procedures involving the use of the animals were in accordance with the Guide for the Care and Use of Laboratory Animals issued by the National Institutes of Health, USA (NIH Publications No. 80–23, 1996), and were approved and monitored by the Ethical Committee of Animal Experiments at the Institute of Neurobiology, Fudan University (Shanghai, China).

### Surgery

#### Implantation of guide cannula for drug administration

Surgical procedures were performed under sodium pentobarbital anesthesia (40 mg/kg i.p.). Rats were restrained in a stereotaxic apparatus (Narishige SN-2, Japan) and implanted bilaterally with guide cannula (stainless steel, 23 gauge), 2.0 mm above the central point of the medial prefrontal cortex (using the coordinates of Panxinos and Watson’s Rats Brain in Stereotaxic Coordinates, 1986; the central point of the medial prefrontal cortex AP +3.5 mm to bregma, ML 0.6 mm to the midsagittal suture line, and V 3.5 mm to the skull surface). The cannula were fixed in place with dental cement and secured with skull screws. A stylus was inserted into the guide cannula to prevent clogging and reduce the risk of infection. Rats were allowed a recovery period of 7 days before behavioral training.

#### Implantation of microelectrode array for spike recording

Rats were initially anesthetized using sodium pentobarbital (40 mg/kg i.p.). Microelectrode array was made by 16 microelectrodes (Formvar-insulated nichrome wires, 35 μm in diameter) in a 2 × 8 configuration with ~200 μm between electrodes and was drivable by turning the screw of microelectrode array. The impedance of each microelectrode was 0.5-1.0 MΩ measured at 500 Hz. Microelectrode array was implanted in the left mPFC (AP 2.5-4.5 mm, ML 0.2-1.0 mm and 2.0 mm below the cortical surface). The microelectrode array was lowered in steps of 0.07 mm every session throughout the recording experiments until the microelectrode tips arrived at 4.0 mm below the cortical surface. The microelectrode array was fixed in place with dental cement and secured with skull screws. A stylus was inserted into the connector of the array to prevent clogging. Rats were allowed a recovery period of 7 days before behavioral training.

### Y-maze spatial delayed alternation task

We used a Y-maze based spatial delayed alternation task to investigate the role of the medial prefrontal cortex in working memory. The Y-maze contained a start box (70 cm long, 15 cm wide and 20 cm high) and two side arms (40 cm long, 15 cm wide and 20 cm high) made up of opaque plastic board, and was placed 70 cm above ground. As shown in Figure 1, the Y-maze had 7 pairs of infrared light emitting diodes, which were used for monitoring the behavioral performance of rats. To start the task, a rat were placed in the start box, and allowed to freely access the left- or right-arm to obtain water. After consuming the water, the rats ran back to the start box, and the start-box door was closed immediately. The rat were ‘imprisoned’ in the start box for 6 seconds (delay period) before it was allowed to make a behavioral choice. The rat was required to choose a side arm opposite to the one visited in the previous trial to get water reward (150 μL). Thus, the rat had to hold in mind, during the forced delay, the spatial information about the arm it had visited in the previous trial, or the arm it was to visit in the present trial. The rat repeated approximately 130 trials and obtained 20 mL water in a daily session.

The rat was given a feedback tone (300 Hz, 0.5 second) after it made an incorrect choice, and no reward was delivered in this case. An error-correction procedure was introduced after an incorrect response choice. That is, the rewarded arm kept unchanged to give the animal a chance to correct its behavioral choice. There were two types of errors in the task: it was possible for the rat not to change its response after a correct choice in the previous trial (win-shift failure), or not adjust its response after an incorrect choice in the previous trial (lose-shift failure). Win-shift strategy reflected the capability of rats to use working memory, whereas the lose-shift strategy reflected the ability of rats to correct errors.

Each trial included four periods: delay, choice, reward and running-back periods (Figure 1). The delay period started when the rat returned to infrared beam #1 in the start box, and lasted 6 seconds. After termination of the delay period, the start-box door dropped down, and the rat ran forwards to make a choice between the two side arms. The choice period was defined as the interval from the dropping down of the start-box door to the rat’s arriving at infrared beam #4 (in the left arm) or #6 (in the right arm). The reward period was defined as the interval when the rat ran from infrared beam #4 or #6 to the end of the arm. The running-back period was defined as the interval when the rat ran from the end of the arm back to infrared beam #1 in the start box.

### Drug administration

To investigate the role of the mPFC in the performance of the spatial delayed alternation task, we locally infused the GABA_A_ receptor agonist muscimol (Sigma, Missouri, USA) into the mPFC to reversibly inactivate this cortical region. Muscimol solution was infused bilaterally (1 μg/μL, 0.5 μL). Saline (0.5 μL) was similarly infused as control. For infusion, rats were held manually, and the stylus was removed from the guide cannula. An infusion needle (30 gauge) was carefully inserted into the guide cannula. The infusion needle extended 2.0 mm from the tip of the guide cannula, targeting at the central point of the mPFC. Infusion was performed at a rate of 0.25 μL/min. The infusion needle was left in place for additional 2 min after completion of the infusion. Bilateral infusions were done simultaneously. Behavioral test was conducted 30 min later.

### Recording of neuronal discharge

Rats received training on the spatial delayed alternation task after recovery from the surgery for microelectrode array implantation. After the animals achieved a criterion of 80% correct performance in two successive sessions, neuronal activities were recorded using neural signal acquiring system (Cyberkinetics, USA). Unit signals were amplified 5000×, band-pass filtered between 0.5 and 7.5 kHz, digitized at 30 kHz, and stored on a personal computer. After a daily recording session was completed, the microelectrodes were advanced gradually in step of 0.07 mm for next-day recording. When the tips of the microelectrode arrived at 4.0 mm below the cortical surface, the recording experiment was terminated.

### Histological procedures

Rats were anesthetized with sodium pentobarbital and transcardially perfused with saline, followed by 4% formaldehyde solution. Brains were removed from skulls, and placed in 10%, 20% and finally 30% sucrose solution for hours till sinking to the bottom of sucrose solution. The brains were sectioned into coronal slices at 40-μm thickness with a cryostat (Leica, Germany). Brain slices were mounted on gelatin-subbed glass slides and stained with neutral red for histological examination of infusion or recording sites.

### Data analysis

#### Isolation of single units

Putative pyramidal cells and interneurons were distinguished by their waveforms and firing frequency [13]. Cells with signal-to-noise ratio of >3 were sampled. Single units were isolated by projecting various spike-waveform parameters. Waveforms located apparently away from the cluster and those with inter-spike interval of <2 ms were excluded, using the offline sorter.

#### Identification of task units

To determine if a cell demonstrates a task-related firing, we compared the firing frequency during a task period (100 ms bin, 50 ms step) with that during the whole trial, using rank-sum test. If p value was <0.01 in three successive bins, we considered the cell as being task-related. For individual cells, we compared the firing frequency in a given task period (100 ms bin, 50 ms step) between left and right trials, using rank-sum test. If p value was <0.01 in three successive bins, the cell was considered as of spatial preference.

#### ROC analysis

Value of receiver operating characteristic (ROC) represents the possibility of neuronal activity tuned by working memory solely by looking at firing frequency [31]. The ROC value ranged in [0.5, 1.0]. While ROC value of 0.5 represents the impossibility of neuronal activity tuned by working memory, ROC value of 1.0 represents the certainty of neuronal activity tuned by working memory. In the present study, we calculated the ROC value of delay-related cells (100 ms bin, 50 ms step). If the ROC value was >0.61 in three successive bins, the delay-related activity was considered as significantly differential [32]. The criterion value (0.61) was determined by calculating the 99th percentage of predicted ROC values in the delay period through shuffling the left and right trials. Then, we drew ROC plot, using MATLAB function (imagesc.m), with working-memory cells on the y-axis against their appearance time of differential firing on the x-axis.

#### Analysis of synchronization

The joint peri-event time histogram (JPETH) quantified the temporal dynamics of interaction between two cells recorded simultaneously, and was calculated as follow:

(1) $${J_{R}}_{i,j}\left(t_{1},t_{2}\right)=\langle S_{i}^{r}\left(t_{1}\right)S_{j}^{r}\left(t_{2}\right)\rangle$$

where i and j are two cells, t_1_ and t_2_ are the t_1_-th and t_2_-th bin of cell i and cell j, respectively; r is the r-th trial, S(t) the count of spikes in time t, and 〈〉 the averaging of S(t) through r trials. To correct for rate modulations, we calculated PSTH predictor as follow:

(2) $$P_{i,j}\left(t_{1},t_{2}\right)=\langle S_{i}^{r}\left(t_{1}\right)\rangle \langle S_{j}^{r}\left(t_{2}\right)\rangle$$

Then, we corrected raw JPETH as follows:

(3) $$J_{i,j}\left(t_{1},t_{2}\right)=\langle S_{i}^{r}\left(t_{1}\right)S_{j}^{r}\left(t_{2}\right)\rangle ‐\langle S_{i}^{r}\left(t_{1}\right)\rangle \langle S_{j}^{r}\left(t_{2}\right)\rangle$$

Finally, the corrected JPETH was normalized by product of standard deviations of spike trains of the two cells as follows:

(4) $$J_{\mathrm{Ni},j}\left(t_{1},t_{2}\right)=\frac{J_{i,j}\left(t_{1},t_{2}\right)}{\sigma_{i}\left(t_{1}\right)\sigma_{j}\left(t_{2}\right)}$$

The value of normalized JPETH ranged in [−1, 1] [33], which was also defined as correlation coefficient. We drew JPETH in sliding time window (75 ms bin, 75 ms step). Because synchronized activity was represented along the main diagonal (from lower left to upper right), we abstracted correlation coefficients along the main diagonal in left and right correct trials, and plotted them in one histogram to facilitate comparison [34].

## Competing interests

The authors declare that they have no competing interests.

## Authors’ contributions

S-TY and J-YP designed experiment. S-TY and YS carried out drug administration experiment; S-TY and QW carried out neuronal discharge recording experiment. S-TY and B-ML analyzed data, wrote manuscript. B-ML conceived of the study. All authors read and approved the final manuscript.
